# Active herpesviruses are associated with intensive care unit (ICU) admission in patients pulmonary infection and alter the respiratory microbiome

**DOI:** 10.3389/fmicb.2024.1441476

**Published:** 2024-08-09

**Authors:** Zhiguang Liu, Chun-jian Qi, Yujia Shi, Tianyu Li, Yuan Fang, Qian Zhang

**Affiliations:** ^1^Department of Respiratory and Critical Care Medicine, The Affiliated Changzhou Second People’s Hospital of Nanjing Medical University, Changzhou, China; ^2^Department of Radiation Oncology, The Affiliated Changzhou Second People’s Hospital of Nanjing Medical University, Changzhou, China; ^3^Genoxor Medical Science and Technology Inc., Shanghai, China; ^4^Changzhou Medical Center, Nanjing Medical University, Changzhou, China

**Keywords:** pulmonary infection, ICU patients, herpesviruses, metagenome, respiratory microbiome

## Abstract

**Background:**

The Herpesviridae family contains several human-related viruses, which are able to establish colonizing and latency in the human body, posing a significant threat to the prognosis of patients. Pulmonary infections represent one of the predominant infectious diseases globally, characterized by diverse and multifaceted clinical manifestations that have consistently attracted clinician’s concern. However, the relationship of herpesviruses on the prognosis of pulmonary infections and the respiratory microbiota remains poorly understood.

**Methods:**

Here, we retrospectively analyzed respiratory samples from 100 patients with pulmonary infection detected by metagenomic next-generation sequencing (mNGS).

**Results:**

Employing mNGS, five herpesvirus species were detected: Human alphaherpesvirus 1 (HSV-1), Human gammaherpesvirus 4 (EBV), Human betaherpesvirus 5 (CMV), Human betaherpesvirus 7 (HHV-7), and Human betaherpesvirus 6B (HHV-6B). Regression analysis showed that the age and positivity of herpesviruses in patients were independently correlated with ICU admission rates. In addition, positivity of herpesvirus was related with increased ICU days and total hospital stay. The herpesvirus-positive group demonstrated markedly higher incidences of co-infections and fungi-positive, predominantly involving *Pneumocystis jirovecii* and *Aspergillus fumigatus*. Analysis of respiratory microbiota revealed a substantially altered community composition within the herpesvirus-positive group, and herpesviruses were significantly positively correlated with the diverse respiratory opportunistic pathogens.

**Conclusion:**

Overall results substantiate that the active herpesviruses in patients with pulmonary infections were significantly associated with high ICU admission rate. Moreover, the herpesviruses promotes the dysbiosis of the respiratory microbiota and an increased proportion of co-infections. These insights could contribute to unraveling the underlying mechanisms connecting active herpesviruses to the progression of severe illnesses.

## Background

Pulmonary infections constitute a leading cause of mortality and morbidity globally, posing a significant threat to both critically ill patients and immunocompromised people (Azoulay et al., 2020). Herpesviruses, such as cytomegalovirus (CMV), Epstein-Barr virus (EBV), herpes simplex virus types 1 and 2 (HSV-1 and -2), and varicella-zoster virus (VZV), can also be the causative pathogens for severe pneumonia, particularly in critical care patients (Saura et al., 2022). These DNA viruses possess the ability to establish latent infections. Previous research has indicated that risk factors for herpesvirus reactivation encompass immunosuppression, invasive mechanical ventilation, extended ICU stays, severe Coronavirus Disease 2019 (COVID-19), and sepsis (Coskun et al., 2017; Saade et al., 2021; Zhang et al., 2021; Bernal and Whitehurst, 2023). Since the high prevalence of herpesvirus infections in the general population, with infection rates reaching 80 to 90%, they are frequently overlooked in clinical diagnosis. Additionally, due to the atypical presentation of viral symptoms, limitations in diagnostic techniques, and ambiguous epidemiology, clinical physicians do not routinely use antiviral therapy, missing the optimal timing for viral treatment. In recent years, the advent of more sensitive and rapid molecular detection methods has revolutionized the landscape of microbiological diagnosis and the identification of specific pathogens. Metagenomic next-generation sequencing (mNGS) technology, capable of sequencing billions of DNA molecules in samples, provides an objective, swift, and unbiased test of pathogen data, emerging as a novel instrument and strategy for the diagnosis and management of lower respiratory tract infections (Diao et al., 2021). This technology has also contributed to an enhanced comprehension and revision of the knowledge pertaining to herpesvirus infections and colonization (Feng et al., 2023; Song et al., 2023; Lu et al., 2024).

The respiratory microbiome is a collection of microorganisms inhabiting the airways of both healthy individuals and those with respiratory illnesses. During the healthy and balanced state, the composition of the lung microbiome helps to induce tolerance and influence the foundational immune response in the lungs (Segal et al., 2016). Dickson et al. (2014) claimed that pneumonia pathogenesis involves a rapid transition from a stable lung microbiome to an imbalanced ecology, marked by decreased microbial diversity, increased microbial burdens, and host inflammation. The advent of mNGS has greatly facilitated microbiome research by offering rapid sequencing, capable of profiling all microbes within the human system through a singular test. Currently, the bulk of mNGS-driven research on herpesvirus concentrates on encephalitis and solid organ transplant patients, with scant studies addressing pulmonary infections. Furthermore, prior studies have not concurrently assessed the reactivation of HSV, CMV, and EBV, nor their associations with patient clinical profiles (Manso et al., 2020; Pan et al., 2022; Xu et al., 2023). Additionally, the interplay between the active herpesviruses and the respiratory microbiomes both the upper and lower tracts in pervasive lung infection cases is yet to be explored. This study seeks to bridge these knowledge gaps by evaluating the clinical characteristics of lung infection patients in relation to these factors.

## Results

### Pathogen spectrum and clinical characteristics

The initial cohort of this study comprised 103 individuals with pulmonary infections, all of whom underwent mNGS testing. Repeated mNGS tests from the same patient were excluded, with the exception of their initial sample. In addition, 3 patients were excluded due to incomplete clinical data, and ultimately 100 patients were included. An illustration of the pathogen spectrum in these patients, as determined by both mNGS and culture methods, is provided in Supplementary Figure 1. This includes 43 bacterial species, 12 fungal species, and 5 viral species, with the most frequently identified pathogens being Human gammaherpesvirus 4 (EBV), *Candida albicans*, and *Haemophilus parainfluenzae* for viruses, fungi, and bacteria, respectively. The detection positivity rate of mNGS was significantly higher than that of conventional culture method (95/100, 95% vs. 22/100, 22%; *P* < 0.0001).

The final clinical diagnosis results of the enrolled patients are shown in Supplementary Table 1, mainly including severe pneumonia, community-acquired pneumonia (CAP), and Corona Virus Disease 2019. The analysis revealed that 15 patients with RNA virus infection had significantly higher ICU admission rates than those without RNA virus detection (14/15, 93.3% vs. 48/85, 6.7%; *P* = 0.0067). Consequently, all 15 patients with diagnosed COVID-19 or H1N1 influenza A were excluded to eliminate the potential confounding effect of RNA viruses on the outcomes. Comparison of clinical data between ICU patients and non-ICU patients indicated no significant difference in metrics such as gender, history of diabetes, history of cardio-cerebrovascular diseases, white blood cell count (WBC), neutrophil count (Ne), and procalcitonin (PCT) (P > 0.05) (Table 1). However, the number of patients aged 60 and above, history of hypertension, the number of patients without underlying diseases, C-reactive protein (CRP), and the Lym % showed significant differences (*P* < 0.05). Notably, the DNA virus positivity rate in ICU patients was significantly higher compared to non-ICU patients (*P* = 0.009). These variables with statistically significant differences were subsequently included as independent variables in a logistic regression analysis (Supplementary Table 2). The analysis demonstrated that patient’s age (≥60 years old) and DNA virus positivity were independent influence factors of patients admission to the ICU (*P* < 0.05).

**TABLE 1:** Demographic and clinical characteristics of ICU and non-ICU patients.

Characteristics	ICU Patients (*n* = 48)	Non-ICU Patients (*n* = 37)	*P-*value
**Age, years**			
≥60, *n* (%)	45 (93.75%)	16 (43.24%)	**<0.0001**
<60, *n* (%)	3 (6.25%)	21 (56.76%)	
**Gender**			
Male, *n* (%)	35 (72.92%)	27 (72.97%)	
Female, *n* (%)	13 (27.08%)	10 (27.03%)	0.995
**Underlying diseases**			
Diabetes, *n* (%)	11 (22.92%)	5 (13.51%)	0.272
Hypertension, *n* (%)	29 (60.42%)	14 (37.84%)	**0.039**
Cerebrovascular disease	7 (14.58%)	2 (5.40%)	0.288
No underlying diseases	4 (8.33%)	14 (37.84%)	**0.001**
**Laboratory tests**			
WBC, × 109/L	11.09 ± 6.44	9.11 ± 3.46	0.102
Neutrophil, × 109/L	8.74 ± 5.03	6.84 ± 3.29	0.054
Lymphocyte, %	10.45 ± 8.35	17.13 ± 8.28	**0.001**
CRP, mg/L	96.75 ± 73.38	51.88 ± 45.83	**0.003**
PCT, ng/L	2.41 ± 6.46	0.24 ± 0.77	0.051
**Detecting DNA viruses by mNGS**			
Positive	28 (58.33%)	11 (29.73%)	**0.009**
Negative	20 (41.67%)	26 (70.27%)	

The bold values indicate statistical significance.

### Analysis of pathogen spectrum in ICU and Non-ICU patients

**FIGURE 1:**
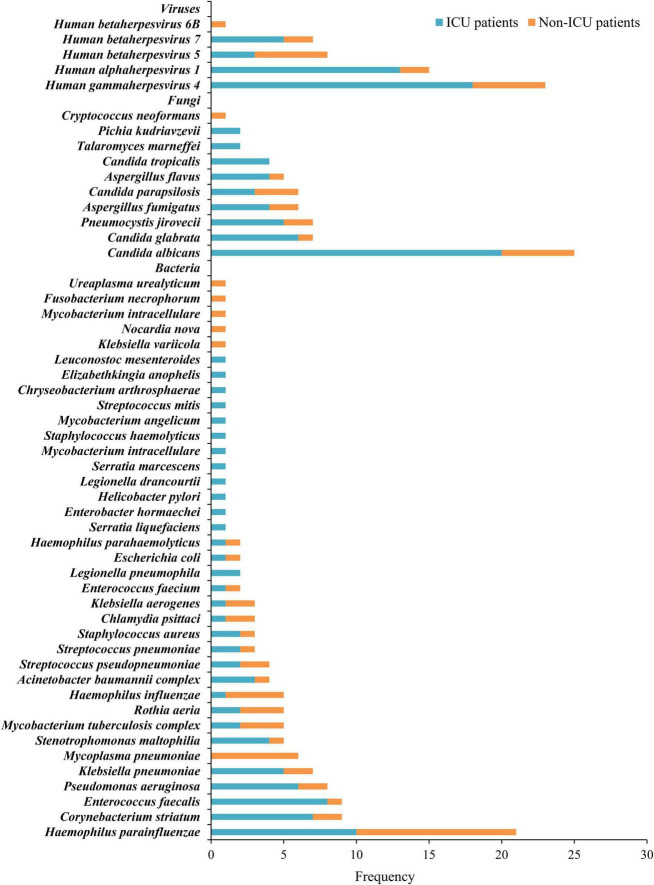
Distribution of bacteria, fungi, and viruses detected by mNGS in ICU and Non-ICU patients.

We utilized mNGS results to establish the pathogen spectrum of ICU and non-ICU patients (Figure 1). The prevalent pathogens among ICU patients were *Candida albicans*, Human gammaherpesvirus 4, Human alphaherpesvirus 1 (HSV-1), *Haemophilus parainfluenzae*, and *Enterococcus faecalis*, while the species with high detection frequency in non-ICU patients were *Haemophilus parainfluenzae*, *Mycoplasma pneumonia*, *Candida albicans*, Human gammaherpesvirus 4, and Human betaherpesvirus 5. Herpesviruses represented the sole viral category detected, encompassing Human alphaherpesvirus 1, Human gammaherpesvirus 4, Human betaherpesvirus 5, Human betaherpesvirus 7 and Human betaherpesvirus 6B.

### The clinical characteristics and prognosis of virus positive patients

Based on whether DNA viruses were identified and reported in the mNGS results, patients with pulmonary infections were stratified into two cohorts: those testing positive for viruses (Virus-Positive, VP) and those testing negative (Virus-Negative, VN). Demographic and clinical characteristics of the remaining patients are summarized in Table 2. The VP group had higher proportions of patients aged over 60 (79.5%) and male patients (74.36%) compared to the VN group. Notably, the proportion of patients with tumors and those with underlying diseases was higher in the VP group compared to the VN group. Laboratory results revealed elevated averages of WBC, Ne, and CRP in the VP group, though these increases were not statistically significant. In terms of treatment and outcomes, the rate of ICU admissions was considerably higher for the VP group (71.80%) versus the VN group (43.48%; *P* = 0.009), and with VP patients also had a noticeably longer ICU stay time than the VN group (20.43 vs. 10.45 days; *P* = 0.173). The average hospitalization duration for the VP group was significantly longer than that of the VN group (20.36 vs. 11.20 days; *P* = 0.029). Additionally, the requirement for mechanical ventilation and the length of its usage trended higher in the VP group, yet these differences did not reach statistical significance (*P* = 0.491 and *P* = 0.265, respectively). Lastly, in-hospital mortality rates were similar between the two groups, with the VP group at 12.82% and the VN group at 6.52%, showing no significant variance (*P* = 0.461).

**TABLE 2:** Demographic and clinical characteristics of VP and VN cases.

Characteristics	VP (*n* = 39)	VN (*n* = 46)	*P*-value
**Age, years**			
≥60, *n* (%)	31 (79.5%)	30 (65.22%)	0.145
<60, *n* (%)	8 (20.5%)	16 (34.78%)	
**Gender**			
Male, *n* (%)	29 (74.36%)	33 (71.74%)	0.787
Female, *n* (%)	10 (25.64%)	13 (28.26%)	
**Underlying diseases**			
Diabetes, *n* (%)	8 (20.51%)	8 (17.39%)	0.714
Hypertension, *n* (%)	19 (48.72%)	23 (50%)	0.906
Cerebrovascular disease	3 (7.70%)	6 (13.04%)	0.424
Cancer	6 (15.38%)	1 (2.17%)	**0.044**
No underlying diseases	6 (15.38%)	12 (26.09%)	0.229
**Laboratory tests**			
WBC, × 109/L	10.69 ± 6.57	10.03 ± 4.33	0.584
Neutrophil, × 109/L	8.99 ± 6.44	8.03 ± 4.43	0.425
Lymphocyte, %	12.46 ± 9.31	14.04 ± 8.78	0.426
CRP, mg/L	82.48 ± 68.91	74.44 ± 65.49	0.586
PCT, ng/L	1.26 ± 3.72	1.69 ± 5.81	0.687
**ICU treatment and prognosis**			
ICU admission, *n* (%)	28 (71.80%)	20 (43.48%)	**0.009**
Duration of ICU stay (days)	20.43 ± 31.68	10.45 ± 6.38	0.173
Use of ventilator, n (%)	11 (28.2%)	10 (21.74%)	0.491
Ventilation time (hours)	443.7 ± 603.80	214.65 ± 186.57	0.265
Total hospitalization time (days)	20.36 ± 26.67	11.20 ± 5.79	**0.029**
In-hospital mortality	12.82%	6.52%	0.461

The bold values indicate statistical significance.

### Comparison of the pathogen spectrum between the VP and the VN group

**FIGURE 2:**
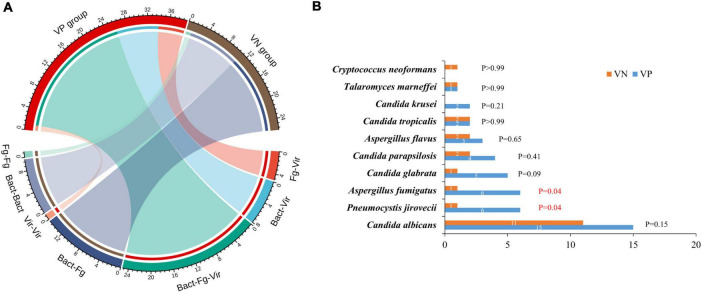
Co-infections detected by mNGS in VP and VN groups. **(A)** Plot of the proportion of each co-infection type in VP and VN patients. Fg, fungi; Bact, bacteria; Vir, viruses. **(B)** Analysis the frequency and differences of fungal detection between VP and VN patients.

The analysis of the pathogen spectrum structure utilizing mNGS results revealed distinct differences between the two groups. In the VP group, all 39 cases exhibited co-infection and no cases of single-pathogen infection were documented. The most prevalent co-infections type involved bacteria-fungi-viruses, accounting for 25 cases, with the remaining cases comprising bacterial-viral and fungal-viral co-infections (Figure 2A). Conversely, 41 patients in the VN group who were detected for potential pathogens, with 26 patients were co-infections, and 15 patients were single-pathogen infections (11 patients with bacterial infection and 4 patients with fungal infection). Bacterial-fungal co-infections were identified in 15 cases and the remaining 11 cases involved single-type co-pathogen infections (Bacterial-bacterial and fungal-fungal co-infections). That is, the VP group exhibited a significantly higher co-infection rate compared to the VN group (100% vs. 63.41%; *p* < 0.0001). It is noteworthy that the prevalence of total fungal detection in the VP group significantly surpassed that in the VN group (76.92% vs. 48.78%; *P* = 0.0094), with the higher detection frequency of *Pneumocystis jirovecii* and *Aspergillus fumigatus* (*P* = 0.044) (Figure 2B).

### The respiratory microbiota characteristics of VP patients

**FIGURE 3:**
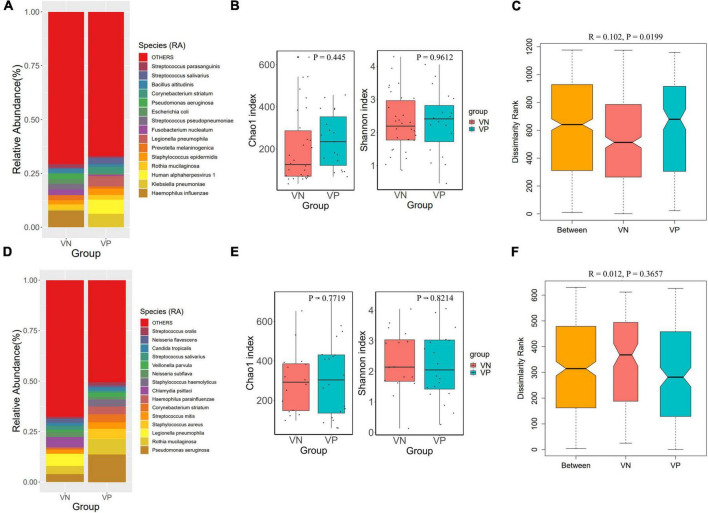
Comparison of respiratory microbiota between VP and VN groups. **(A)** Species-level species composition chart of BALF samples. **(B)** Alpha diversity (Chao 1 and Shannon indexes) of each group of BALF samples. **(C)** ANOSIM boxplot of BALF samples. **(D)** Species-level species composition chart of sputum samples. **(E)** Alpha diversity (Chao 1 and Shannon indexes) of each group of sputum samples. **(F)** ANOSIM boxplot of sputum samples.

To explore the relationship between herpesviruses and the respiratory tract microbiome in cases of pulmonary infections, the metagenomic data from VN and VP patients were compared across different sample types. The proportional distribution of microbial abundance in the BALF samples is shown in Figure 3A. At the species level, *Haemophilus influenzae*, *Klebsiella pneumoniae*, Herpesvirus 1, and *Rothia mucilaginosa* were prominent in the microbiota of both cohorts. In contrast to the VN group, the VP group exhibited a reduced abundance of *Haemophilus influenzae* and an increased abundance of *Klebsiella pneumoniae*. The alpha diversity analysis indicated no notable differences in microbial richness or diversity between the groups in the lower respiratory tract (Figure 3B). Additionally, ANOSIM analysis based on Bray-Curtis distance revealed significant disparities in the microbial community composition between the VN and VP groups (*R* = 0.102, *P* = 0.0199) (Figure 3C).

Figure 3D displays the percentage distribution of microbial abundance in sputum specimens. At the species level, *Pseudomonas aeruginosa*, *Rothia mucilaginosa*, *Legionella pneumophila*, and *Staphylococcus aureus* were predominant in both groups. Compared to the VN, the VP had a higher abundance of *Pseudomonas aeruginosa*, but a lower abundance of *Legionella pneumophila*. Alpha diversity analysis showed that there were no significant differences between the two groups in the upper respiratory tract microbiome (Figure 3E). Results of ANOSIM analysis based on Bray-Curtis distance indicated a difference in microbial community structure between the groups, although this variation was not statistically significant (*R* = 0.012, *P* = 0.3657) as depicted in Figure 3F.

### Analysis of the correlation between herpesvirus and high abundance species

**FIGURE 4:**
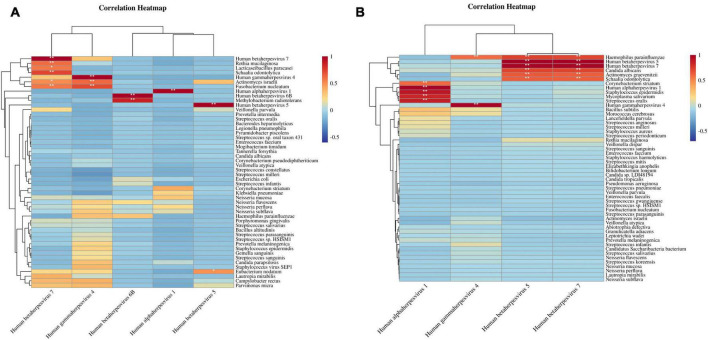
Correlation heatmaps of herpesviruses and high-abundance species. **(A)** Correlation between herpesviruses and top 50 high-abundance species in BALF samples. **(B)** Correlation between herpesviruses and top 50 high-abundance species in sputum samples. The correlation coefficient ranges from [–1, 1], * denotes the *P*-value < 0.05 of the significance test of the correlation coefficient, ** denotes the *P*-value < 0.01.

Further analysis explored the association between herpesviruses and the top 50 most abundant species in both sample types. Spearman correlation coefficient assessments shown that in BALF samples, herpesviruses were markedly positively correlated with several species, including *Actinomyces israelii*, *Rothia mucilaginosa*, *Fusobacterium nucleatum*, *Methylobacterium radiotolerans*, *Lacticaseibacillus paracasei*, *Schaalia odontolytica*, and *Eubacterium nodatum*, as illustrated in Figure 4A. Correspondingly, in sputum samples, herpesviruses exhibited a substantial positive correlation with different species, such as *Haemophilus parainfluenzae*, *Candida albicans*, *Corynebacterium striatum*, *Actinomyces graevenitzii*, *Staphylococcus epidermidis*, *Mycoplasma salivarium*, *Schaalia odontolytica*, and *Streptococcus oralis* (Figure 4B). Noteworthy is the distinctive positive correlation of *Haemophilus parainfluenzae* with three herpesviruses (EBV, CMV, and HHV-7).

## Discussion

Herpesvirus infections may negatively influence patient prognosis, and its role as a pathogen has been extensively studied. Nevertheless, the clinical implications of viral activation have not been adequately addressed. Our research utilized the more sensitive mNGS method to analyze respiratory specimens from patients with lung infections. We compared individuals exhibiting the active herpesviruses with those without, thus offering a detailed overview of the lung microbiome in patients experiencing herpesvirus activation.

This study observed a notably high proportion of severe cases in patients with pulmonary infections, therefore early implementation of empirical anti-infective treatments may compromised the detection capability of traditional culture methods, which rely on the growth of pathogens on culture medium. The limitation was particularly acute for hard-to-culture or slow-growing organisms, such as anaerobes and filamentous fungi, thereby reducing the sensitivity of conventional detection techniques (Gu et al., 2019). The present findings indicate that the mNGS technology markedly outperformed culture methods in both the positive detection rate and the diversity of identified pathogens. Therefore, when facing the diagnosis and treatment of unknown, difficult, and critically ill infected patients, we encourage doctors to strengthen the clinical application of mNGS technology in a reasonable manner.

Herpesvirus reactivation is frequently observed in patients with severe lung infections. Upon analyzing the pathogen spectrum in all patients, including those in ICU, herpesviruses emerged as the sole DNA viruses detected, predominantly EBV and HSV-1. Excluding CMV, these viruses were more commonly identified in sputum samples, possibly because CMV can colonize the lower respiratory tract in healthy individuals (Ross et al., 2010). A prospective cohort study showed that HSV was isolated in 58 (16%) of 361 lower respiratory tract specimens from critically ill patients (Bruynseels et al., 2003). Luyt et al. (2022) determined that HSV is a prevalent pathogen in hospital-acquired and ventilator-associated pneumonia (HAP/VAP). Similarly, 21% of ICU patients on mechanical ventilation were diagnosed with HSV-1 bronchopneumonia (Luyt et al., 2007). Although HSV can be detected sporadically in airway samples of critically ill patients, actual HSV pneumonia is rare. Active HSV-1 is considered clinically significant when a patient shows severe manifestations such as systemic skin symptoms, hepatitis or encephalitis, and worsening respiratory functions and lung lesions. EBV infecting over 95% of the global population and transmitted primarily via saliva, often results in mild or asymptomatic infections. Research has revealed that the reactivation of EBV is associated with long-haul COVID syndrome (Vojdani et al., 2023). CMV-related colitis, retinitis, hepatitis, and pneumonia have all been reported, most commonly in immunocompromised patients. Active CMV is frequent found in respiratory secretions of critically ill patients receiving mechanical ventilation and in severe COVID-19 patients. Researches in the field of herpesviruses has reported that reactivation of CMV, HSV-1, and EBV are associated with a 28-day increase in all-cause mortality in patients with severe pneumonia (Huang et al., 2023). It is worth mentioning that studies of individual herpesviruses have shown that the presence of HSV-1 is associated with increased 90-day mortality in patients with severe pneumonia (Liu et al., 2023). However, Xu et al. (2023) reported that HHV-7 positivity was not an independent risk factor for 28-day mortality. Due to the lack of actual herpesvirus pneumonia patients, the causal relationship between CMV and EBV alone with increased ICU stay and mortality is still unclear. In this regard, we showed that the positive viral group had a higher proportion of underlying disease patients, but there was no significant difference. As our enrolled patients lacked information on routine medication, comparisons of immunosuppressive status were not made. Notably, the VP group experienced higher ICU admission rates and longer hospital stays, supporting previous findings.

Severe patients with viral co-infections tend to be overlooked, particularly those infected involving latent viruses. Compared to conventional methods, mNGS simplifies clinical testing for diagnosing pulmonary co-infections. Hou et al. (2022) used mNGS to analyze patients with herpes simplex pneumonia and co-infections, revealing that patients with HSV-1 co-infections exhibited more pronounced inflammatory responses than those with HSV-1 alone. *Pneumocystis jirovecii* emerged as the most commonly identified co-infecting species (Hou et al., 2022). Our finding found that patients positive for herpesvirus were more prone to co-infections, especially with fungal pathogens, notably *Pneumocystis jirovecii* and *Aspergillus fumigatus*. Analogous to herpesviruses, *Pneumocystis jirovecii* may silently inhabit the lungs of patients. *Pneumocystis jirovecii* and CMV in pulmonary co-infections have been widely reported (Yu et al., 2017; Korkmaz Ekren et al., 2018). A recent investigation by Rucar et al. (2024) explored the correlation between mortality and other herpesviruses (EBV, HSV-1 and -2, VZV) in patients with Pneumocystis pneumonia (PCP), showing that co-positivity with EBV and CMV in patients’ BALF substantially elevates the risk of mortality. *Aspergillus fumigatus* has been identified as a significant cause of pneumonia in individuals with compromised immune systems (Afessa and Peters, 2006; Patel and Greenberger, 2019). The presence of HSV-1 was correlated with a higher incidence of *Aspergillus fumigatus* detection, amounting to 12.0% in these patients (Hou et al., 2022). Another retrospective study showed CMV was the most common co-pathogen in mixed infections of invasive pulmonary *aspergillosis* patients (Shi et al., 2023). In addition, the coexistence of herpesvirus with *Aspergillus fumigatus* and *Pneumocystis jirovecii* has also been found in children (Zhang et al., 2023). The presence of herpesvirus may alter the respiratory tract’s microenvironment in patients, potentially leading to microbiota dysbiosis and subsequent modulation of immune responses that increases vulnerability. To investigate this hypothesis, we conducted an analysis of both upper and lower respiratory tract microbiota. The results indicated that the presence of herpesviruses affects the lower respiratory microbiota. These results are in agreement with the perspectives presented by Liu et al. (2023). Furthermore, we observed differences in the prevalence of certain prevalent opportunistic pathogens, including *Klebsiella pneumoniae* and *Pseudomonas aeruginosa*, between the groups. This suggests that herpesvirus may create a conducive environment for the proliferation of these potential pathogens. Subsequent correlation analysis between the microbiome reinforced this notion, revealing that herpesviruses exhibited significant positive correlations with numerous opportunistic pathogens, particularly within the analyses of the upper respiratory tract. These included pathogens commonly encountered in ICU patients, such as *Haemophilus parainfluenzae*, *Candida albicans*, and *Corynebacterium striatum*. Research focusing on sepsis patients demonstrated that patients testing positive for viruses like CMV, EBV, and HSV were found to have an elevated risk of succumbing to secondary fungal infections and conditional pathogenic bacterial infections (Walton et al., 2014).

Current research on the active herpesviruses predominantly involves ICU patients. Once diagnosed with herpesvirus pneumonia, ICU patients will face poor prognosis, with a mortality rate as high as 60–63% (Simoons-Smit et al., 2006). Thus, early identification of herpesvirus pneumonia is crucial. Clinical treatment protocols should incorporate the administration of antiviral medications like acyclovir, guided by clinical manifestations, corroborated findings, and evidence of cytopathological alterations in bronchoalveolar samples (Pata and Datar, 2023). This study examined pulmonary infection in patients and demonstrated that herpesvirus positivity was strongly associated with admission to the ICU, prolonged ICU stays, and an increased incidence of opportunistic pathogen co-infections. It is recommended that clinical surveillance for herpesvirus be intensified in patients with pulmonary infections through the utilization of various diagnostic modalities, including nucleic acid amplification tests, mNGS, viral culture, and antigen detection assays. Careful monitoring of immune markers and pathogenic spectrum in patients who test positive is essential to prevent worsening of the condition and polymicrobial infections.

One limitation of this research is the inability to compare the viral abundance and clinical data of ICU herpesvirus positive patients, both before and after their admission to the ICU. Thus, it remains undetermined whether active herpesviruses are merely an epiphenomenon of ICU admission, or contributed to the development of severe disease, this necessitates addressing in a distinct prospective study. In addition, whether the active herpesviruses in pneumonia patients merely affects their immune status or exerts a definitive pathogenic capability necessitates additional investigation. For instance, utilizing PCR technology for continuous monitoring could facilitate the determination of the relationship between pulmonary viral load and patient prognosis, as well as immune status.

## Conclusion

This study demonstrates that HSV-1, EBV, and CMV are the most frequently activated DNA viruses in the lungs of patients with pulmonary infections. Such activation links to an uptick in ICU admissions and extended hospital stays, pointing to these viruses may be potential markers of adverse outcomes in lung infection cases. Moreover, the active herpesviruses correlate with alterations in the respiratory microbiota composition, and the resultant synergistic interactions with various opportunistic pathogens could promote concurrent fungal and bacterial infections.

## Materials and methods

### Study design and sample collection

Patients with suspected pulmonary infections were selected from the Department of Pulmonary and Critical Care Medicine at the Affiliated Changzhou Second People’s Hospital of Nanjing Medical University, recruited between October 2022 and January 2024. This study complied with the Declaration of Helsinki and was approved by the Ethics Committee of the Affiliated Changzhou Second People’s Hospital of Nanjing Medical University. Due to the retrospective nature of the study and as no identifiable patient information was included in this manuscript, the need for consent was waived. A total of 105 patients were investigated by reviewing their electronic medical records. Enrollment criteria for suspected pulmonary infection included: (i) imaging evidence of pulmonary exudation; (ii) clinical symptoms such as fever, cough, expectoration, and respiratory failure characteristic of pulmonary infections. Additional inclusion criteria were: (i) consent from pneumonia patients to collect respiratory tract samples, with subsequent pathogen detection via mNGS and culture; (ii) the quality inspection and samples testing process met the standards of mNGS; (iii) availability of complete clinical data. Patients failing to meet these criteria were excluded from the study.

### Metagenomic DNA/RNA sequencing and analysis

The total DNA of the BALF and sputum samples was extracted using the Tiangen Magnetic DNA Kit (Tiangen, China). The DNA libraries were constructed using a multistep process: DNA fragmentation, end-repair, add A-tailing, adapter ligation, and PCR amplification utilizing the NEB Next^®^ Ultra™ DNA Library Prep Kit for Illumina^®^ sequencing. Quality assessment of the DNA libraries was conducted using an Agilent 2100 bioanalyzer, and library concentration was measured with the Qubit 2.0 Fluorometer. The qualified libraries were sequenced using the Illumina Next-seq platform. Individual patients who were clinically suspected to be infected with RNA virus underwent mNGS-RNA testing simultaneously. RNA was extracted using the RNA Easy Fast Kit (Qiagen, China) according to the manufacturer’s instructions. The RNA were pooled and reverse transcribed into cDNA using HiScript^®^III 1st Strand cDNA Synthesis Kit (Vazyme, China), followed by library construction and sequencing as above. Raw sequence data were splitted using bcl2fastq2 software. Adaptor sequences and low-quality bases were trimmed using Trimmomatic software to yield high-quality, effective sequences. Human host sequences were filtered out utilizing bowtie2 software, leaving non-human sequences available for alignment with the microbial genome database. This database was constructed by curating standard microbial nucleic acid sequences from public repositories. To compare the abundance of different species within the same sample, the number of reads was normalized by the length of the species genome to calculate their “Reads Per Kilobase (RPK).” This metric was employed to calculate the species’ relative abundance. Alpha diversity measured by the Shannon and Chao1 index was used to assess the complexity of taxonomic diversity. Differences in the microbial composition among different samples were analyzed using Analysis of Similarities (ANOSIM) based on the Bray-Curtis dissimilarity algorithm. Spearman correlation analysis was used to analyze the microbial interactions.

### Criteria for a positive mNGS result

The detection list was generated by applying a threshold criterion, where thresholds were defined based on the number of reads that were stringently mapped to bacterial, mycoplasma, chlamydia, DNA virus or fungal species-with a minimum requirement of three reads. For the Mycobacterium tuberculosis complex (MTC), ≥ with a minimum requirement of one read. RNA virus was defined as 1 or more reads on the genome being covered.

### Laboratory microbial culture and viral nucleic acid detection

According to the culture procedures for bacteria and fungi in the diagnostic laboratory of the Changzhou NO.2 People’s Hospital, the sample culture was performed using routine isolation media, including blood agar, mackcoys agar, chocolate agar, and Mueller-Hinton agar. Plates were incubated at 37°C in 5–10% CO_2_ for 24–48 h using Heal Force HF90 incubator (Heal Force Bio-Meditech Co., Ltd., China). The strains were then isolated and identified the species using the VITEK-2 Compact Instrument (BioMérieux, France). Upper respiratory tract specimens were collected from both nasopharyngeal or oropharyngeal swabs by trained medical staffs. Subsequently, according to the manufacturer’s instructions (Bioperfectus, China), SARS-CoV-2 was diagnosed using real-time polymerase chain reaction (RT-PCR).

### Statistical analyses

Continuous variables were described by medians, and categorical variables were expressed as counts and percentages. Chi-square test or Fisher’s exact test were performed for categorical variables and t-tests for continuous variables using GraphPad Prism 7 software. Binary Logistic regression analysis was performed using SPSS 25.0 software. *P*-value < 0.05 was considered to represent a significant difference.

## Data availability statement

The datasets presented in this study can be found in online repositories. The names of the repository/repositories and accession number(s) can be found below: https://www.ncbi.nlm.nih.gov/, PRJNA1097807.

## Ethics statement

The studies involving humans were approved by the Ethics Committee of the Affiliated Changzhou Second People’s Hospital of Nanjing Medical University. The studies were conducted in accordance with the local legislation and institutional requirements. The participants provided their written informed consent to participate in this study.

## Author contributions

ZL: Funding acquisition, Formal analysis, Writing – original draft. C-jQ: Writing – review and editing. YS: Formal analysis, Visualization, Writing – original draft. TL: Writing – original draft, Methodology, Software. YF: Writing – original draft, Data curation. QZ: Conceptualization, Funding acquisition, Writing – review and editing.
