# Supplementary material for: Active herpesviruses are associated with intensive care unit (ICU) admission in patients pulmonary infection and alter the respiratory microbiome
Source: Front Microbiol. 2024 Aug 9;15:1441476. doi: 10.3389/fmicb.2024.1441476 (PMC11342977; doi:10.3389/fmicb.2024.1441476)
Supplement: Supplementary file 2 [file Data_Sheet_1.docx]

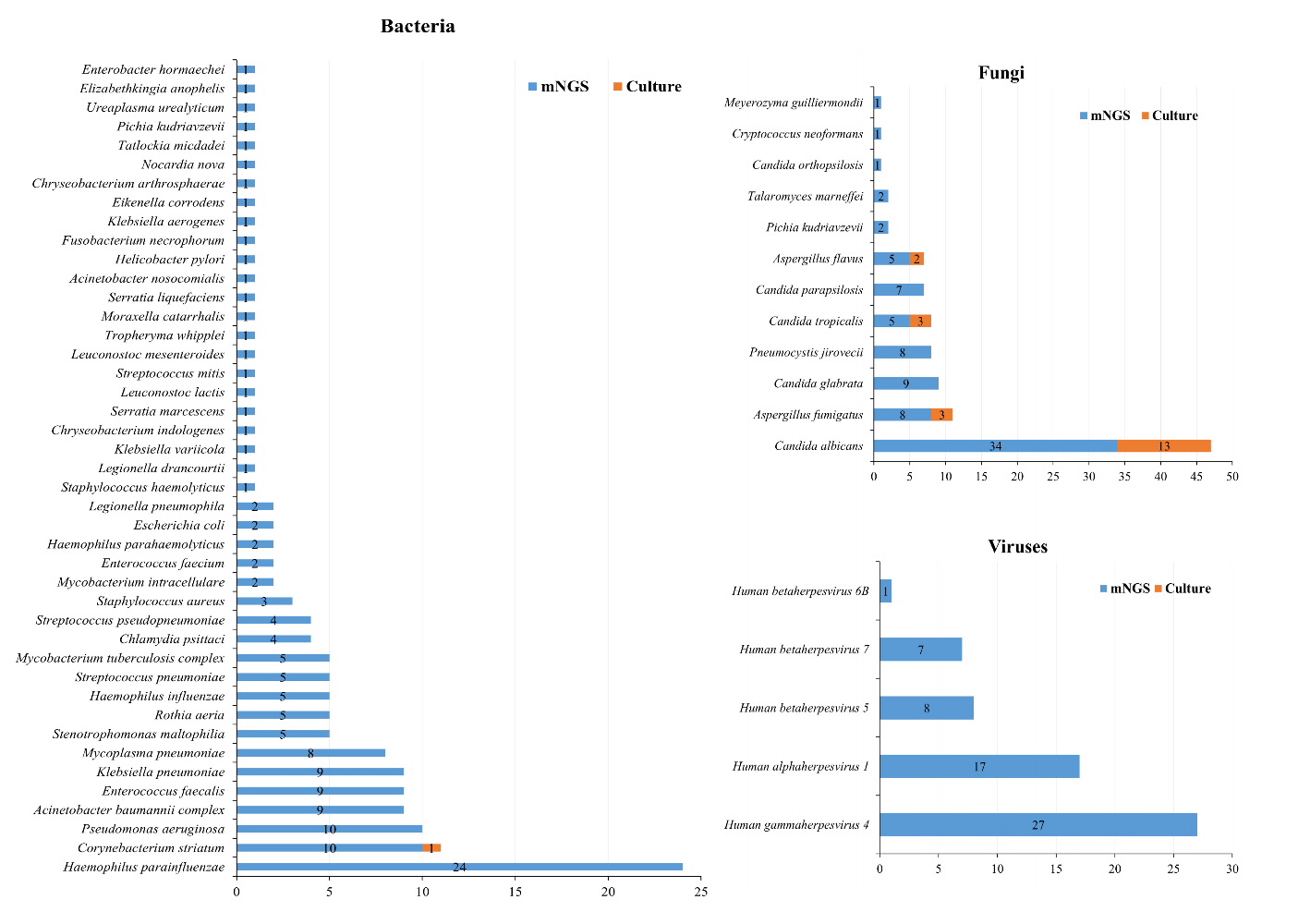


**Supplementary Figure 1** The pathogen spectrum of the final enrolled patients with pulmonary infection.

**Supplementary Table 2** Logistic regression analysis of patients with pulmonary infection admitted to ICU.

| **Variable** | ***β*** | ***SE*** | ***Wald X^2^*** | ***P*** | **OR (95%CI)** |
| --- | --- | --- | --- | --- | --- |
| ＜60 years |  |  |  |  |  |
| ≥60 years | 2.228 | 0.818 | 7.422 | **0.006** | 9.284 (1.869-26.673) |
| Detecting DNA viruses by mNGS |  |  |  |  |  |
| Negative |  |  |  |  |  |
| Positive | 1.228 | 0.607 | 4.097 | **0.043** | 3.415 (1.040-11.219) |
| Underlying diseases | 1.395 | 0.922 | 2.290 | 0.130 | 4.034 (0.663-24.559) |
| Hypertension | 0.300 | 0.689 | 0.002 | 0.965 | 1.031 (0.267-3.976) |
| Lymphocyte, % | -0.009 | 0.038 | 0.58 | 0.809 | 0.991 (0.920-1.067) |
| CRP, mg/L | 0.009 | 0.007 | 1.969 | 0.161 | 1.009 (0.996-1.022) |
